# On Congenital Defects of the Enamel

**Published:** 1893-10

**Authors:** Otto Zsigmondy

**Affiliations:** Vienna, Austria


### ON CONGENITAL DEFECTS OF THE ENAMEL.[1]

BY DR. OTTO ZSIGMONDY, VIENNA, AUSTRIA.

THAT striking anomaly in enamel-development known as "erosion" or "atrophy" occurs quite frequently. In teeth so affected the coating of enamel is unequally distributed. In the lowest grades of the affection, superficial pits of greater or less depth are found at isolated points in the otherwise apparently normal enamel. These pits may be isolated, or they may occur in rows. When the pits are confluent, furrows are developed which may embrace the crown like a ring. At times several of such furrows are found arrayed in series one above the other (furrows or wavy enamel). In other cases the enamel appears as if sown with small pits (honeycombed teeth of the English). In still other cases the enamel seems to be entirely absent at certain points, and the dentine itself comes to the surface (called *érosion en échancrure* and *érosion en nappe* by French writers if the defect affects the free edge of the cutting-surface or the front teeth to a greater or less extent).

The layer of enamel lining the pits possesses a rough, uneven surface, a more or less yellowish or brownish color, and lacks the proper polish and normal transparency, while the enamel in the immediate neighborhood of the spots has all the characteristics of the perfect tissue. In those teeth in which the dentine is exposed or covered by a thin layer of enamel only, the enamel of the normal portion of the tooth stands out in the form of an annular swelling, a circumstance which has influenced certain authors to speak of the thickening of the enamel at those points. But, as can readily be seen in sections of such teeth, a true increase in the thickness of the

---

[1] Abstract from a paper read by Dr. Zsigmondy, World's Columbian Dental Congress, August, 1893.

layer of enamel above the normal never occurs. While the surface of the enamel is seen on cross-sections to be interrupted, dipping into the pits and furrows, the margin of the dentine seems unchanged to the naked eye. Thus portions of different thickness are found in the layer of enamel. Spots entirely devoid of enamel are found but very rarely. Spots entirely deprived of enamel, as a rule, soon succumb to caries, especially as the dentine of such teeth possesses a faulty structure conducive to the progress of the affection at those points where no enamel is present. It is characteristic of the defects in question that they are always symmetrical, so that corresponding teeth in the upper and lower jaws on both sides are similarly affected. The defects occur in the teeth whose development corresponds to the same period of time, and in the teeth they are limited to such portions as correspond in degree of development. The situation of the defects and their distance from the lateral surface of the crown accordingly vary in the different orders of the teeth. At times, if the interference with the development occurred at a very early period and no interruption in normal development took place later, it is only the first four molars, the first of the permanent teeth to harden, which show signs of abnormality. The defects are then found at the apices of the cusps only, which in consequence seem as if worn down. If the interruption of normal development occurs at a later period, when the formation of the enamel of the cusps of the first molars is further advanced, these teeth show the defect more in the direction of the root. In this case, besides the first molars, the central incisors likewise show defects in the enamel of the teeth which come next in point of time of calcification.

If the disturbance be repeated once or oftener, a corresponding series of furrows or pits will be found in the enamel of the teeth which were in process of calcification at the time of interruption of the development. If a furrow be found in the first molar near the edge, an analogous defect will appear half-way up on the crown of the central incisor. The cuspid will show the defect nearer the point of the cusp, while the first molar and the teeth which undergo calcification later will be free from defect. The lateral incisor of the upper jaw, it should be remarked, differs from the corresponding tooth of the lower jaw, whereas its calcification follows that of the central incisor of the lower jaw and precedes that of the cuspid. Calcification in the upper jaw proceeds as follows:

1. The central incisor.
2. Cuspid.

3. Lateral incisor.

This noteworthy circumstance, as yet inadequately studied, determines why we find the lateral incisor developed almost normally, while defects are apparent in the enamel of other teeth of the upper jaw. I shall not stop to consider these phenomena more at length at present; the necessary data will shortly be given in a publication by my friend G. Cunningham, of Cambridge, England.

The first molar is but rarely the seat of typical defects, the second molar still more rarely, and no instance of such defect has ever been observed in the two last molars. Certain writers maintain that the milk-teeth never exhibit typical defects. This, however, is a mistake. Temporary teeth are occasionally observed which resemble permanent cuspids and molars in defects of the enamel, and whose internal structure also shows the discontinuous lines which are characteristic of the teeth in question.

The question of the etiology of these deformities has often formed the subject of lively discussion. Seeing that the anomaly is hardly ever limited to a single tooth, as would be the case if the cause were purely local, but involves in the majority of cases the entire denture, this circumstance compels us to seek for the cause of the anomaly in general diseases of the organism whose effect as regards the other tissues of the body has vanished, while its influence has become permanent in the teeth.

Rachitis, scrofulosis, syphilis, the exanthemata, convulsions, meningitis, grave attacks of suffocation, as, for example, from whooping-cough in early life, have been the alleged causes of the disturbances to the normal development of the teeth in their follicles. In view of the literature dealing with the subject, it is rather astonishing that the microscopic examinations of teeth so affected should have been almost entirely neglected, as this must form the basis for the solution of the question of the cause of the deformities. A point of capital importance is the evidence that the disturbance in development can also be observed in the dentine. The latter is not uniformly calcified in all its parts. We observe in sections of the same, at points corresponding to the fundamental strata, well-marked lines which consist of interglobular spaces arranged in rows. These lines are sections of strata in which incomplete calcification has taken place.

The few data contained in the literature bearing on the structure of teeth with defective enamel are presented by C. Wedl, in his "Pathology of the Teeth;" R. Baume, "The Defects of the Solid Substance of the Teeth," Leipsig, 1882; A. Walkhoff, "A Contribution to the Theory of theLines of Contour and to the Physiology of the Dentine," *Deutsche Monatsschrift für Zahnheilk.*, 1885, p. 576; and Charles S. Tomes, "A System of Dental Surgery," third edition, 1887.

We are indebted to Frank Abbott ("Congenital Defects in Enamel," *Dental Cosmos*, 1891, p. 605) for more extensive data on the structure of imperfectly-developed enamel. The enamel-prisms are characterized according to this author by their wavy form, and are interrupted by delicately-marked concentric striations.

Penetrating the enamel at varying heights are seen numerous pear-shaped prolongations of the dentinal canaliculi, some extending nearly to the surface; these spaces contain protoplasm, and are stained a deep-violet color by chloride of gold. At several points layers of enamel with granular pigment are seen, where the enamel has not been thoroughly calcified. In one case Abbott observed an anomalous outgrowth of enamel upon the normal enamel. In another case an originally deficient enamel was found on which was deposited a normal one.

The question whether the enamel formed coincidently with the interglobular spaces in the lamina of the dentine may not also show traces of interruption to the normal development finds no answer in the literature of the subject. Researches undertaken with this object have shown that an analogy between the lines of the dentine and the tissue of the enamel really exists.

Longitudinal section of a tooth affected with the simplest form of defect in the enamel, a furrow enveloping the crown, shows that the layer of enamel in the situation of the defect becomes gradually but progessively thinner in a direction from the apex of the crown to the root, until it reaches the deepest point of the depression, where the enamel becomes reduced to an insignificant layer varying in amount in different cases. The enamel rapidly regains its normal thickness from the deepest point of the depression in the direction of the root. The external boundary-line of the enamel gradually approaches the surface of the dentine, whence it suddenly turns sharply outward. If the section be sufficiently thin, we observe a delicate but well-marked line, broken here and there, which traverses the whole extent of the enamel as far as the surface of the dentine, and in direct continuation of the first portion of the line limiting the defect and running in the same direction. This line makes an angle of fifteen to thirty degrees with the surface of the dentine. Its direction is thus as a whole the same as that of the brownish parallel bands of Retzius, which frequently occur in considerable numbers in the adjacent enamel. In cross-sections we see the line running parallel to the surface of the dentine and surrounding the entire crown of the tooth. The position of the line is therefore such that it is to be regarded as the section of a plane which divides the portion of enamel first from that deposited subsequently. It is probably correct to assume that it marks the limit of calcification of that portion of the enamel deposited at the time when the cause of the disturbance of development made itself felt. If a section of a normally-developing tooth be examined and the lines of demarcation between the latest deposit of enamel observed, it will be found that the layer of enamel reaches its greatest thickness at the free extremity, at the cutting-edge, or at the summit of the crown-points where calcification commences, and becomes gradually attenuated towards the roots.

The enamel-prisms corresponding to the first portion deposited are already developed to a considerable extent as regards length, while the prisms of the remaining part are not so far advanced, and in that portion still farther distant from apex of the crown calcifications were observed to have only begun in the prisms. The enamel-cells in the region of transition between the internal and external epithelium of the enamel have at this period not yet begun the formation of enamel. If the disturbance to the nutrition of the enamel occurs at this time, those cells of the enamel which have produced the prisms already calcified to a considerable extent are destroyed, hence the further development of the prisms becomes impossible. Those prisms belonging to the enamel situated nearer the crown of the tooth not being developed to the same extent, while suffering some interruption in development, nevertheless continue to progress after the disturbing influence has ceased to make itself felt, and may in many cases attain their normal size. The prisms in the region of the neck of the tooth in which calcification has not yet begun at the time of activity of the disturbance of course suffer no interruption to development.

The line of interruption above described is always found in all forms of defective enamel. Where the surface of the crown shows two or more furrows, the layer of enamel is seen on longitudinal section to be separable, with a corresponding number of strata, by lines extending from the furrows to the surface of the dentine. In those cases, also, where the defect consists of only a pit-like depression, we find the line of interruption on longitudinal section. It is exactly like those cases where the crown is embraced by a furrow on cross-section; it is noticed that the line stretches from the bottom of the pit on both sides and surrounds the entire crown.

I cannot leave the subject without making a few remarks on the nomenclature which has been used to describe the defects under consideration. Terms which are appropriate for a great many cases, such as "wavy enamel," "honeycombed teeth," "furrowed teeth," and similar expressions, are not suitable in every case. The descriptive appellations "syphilitic" or "rachitic" teeth are to be rejected, because it is doubtful whether the diseases are the real cause of the defects, and if they are a cause they can only be so in a small proportion of cases. The term "atrophy" is likewise incorrect. The expression "erosion" is no less to be rejected. Erosion signifies loss of substance by mechanical force. The term erosion is properly applied to the notch-like grooves found on the necks of teeth, and which have been produced by the use of gritty tooth-powders, etc. I shall take the liberty of offering the following: Where individual organs or parts of organs are defectively developed because of external or internal noxæ, pathological anatomists are wont to employ the term hypoplasia to express that condition. We may accordingly speak of a hypoplasia of the enamel.

May I be permitted to express the hope that this term may soon find a place in the literature of the subject?

